# Moderate Elevation of Homocysteine Induces Endothelial Dysfunction through Adaptive UPR Activation and Metabolic Rewiring

**DOI:** 10.3390/cells13030214

**Published:** 2024-01-24

**Authors:** Barun Chatterjee, Fabeha Fatima, Surabhi Seth, Soumya Sinha Roy

**Affiliations:** 1 CSIR-Institute of Genomics & Integrative Biology, New Delhi 110025, India; b.chatterjee@igib.res.in (B.C.); fabeha.fatima@igib.res.in (F.F.); surabhi.seth@igib.res.in (S.S.); 2 Academy of Scientific & Innovative Research, Ghaziabad 201002, India

**Keywords:** homocysteine, endothelial cell, angiogenesis, zebrafish, ER stress, actin cytoskeleton, mitochondria, glycolysis, TCA cycle

## Abstract

Elevation of the intermediate amino acid metabolite Homocysteine (Hcy) causes Hyperhomocysteinemia (HHcy), a metabolic disorder frequently associated with mutations in the methionine-cysteine metabolic cycle as well as with nutritional deficiency and aging. The previous literature suggests that HHcy is a strong risk factor for cardiovascular diseases. Severe HHcy is well-established to correlate with vascular pathologies primarily via endothelial cell death. Though moderate HHcy is more prevalent and associated with an increased risk of cardiovascular abnormalities in later part of life, its precise role in endothelial physiology is largely unknown. In this study, we report that moderate elevation of Hcy causes endothelial dysfunction through impairment of their migration and proliferation. We established that unlike severe elevation of Hcy, moderate HHcy is not associated with suppression of endothelial VEGF/VEGFR transcripts and ROS induction. We further showed that moderate HHcy induces a sub-lethal ER stress that causes defective endothelial migration through abnormal actin cytoskeletal remodeling. We also found that sub-lethal increase in Hcy causes endothelial proliferation defect by suppressing mitochondrial respiration and concomitantly increases glycolysis to compensate the consequential ATP loss and maintain overall energy homeostasis. Finally, analyzing a previously published microarray dataset, we confirmed that these hallmarks of moderate HHcy are conserved in adult endothelial cells as well. Thus, we identified adaptive UPR and metabolic rewiring as two key mechanistic signatures in moderate HHcy-associated endothelial dysfunction. As HHcy is clinically associated with enhanced vascular inflammation and hypercoagulability, identifying these mechanistic pathways may serve as future targets to regulate endothelial function and health.

## 1. Introduction

Hyperhomocysteinemia (HHcy) is a metabolic abnormality characterized by unusually high plasma levels of Hcy, a sulfur-containing non-proteinogenic amino acid produced during the conversion of dietary methionine to cysteine. This pathological condition can arise either due to a nutritional deficiency of folate and B vitamins, required as cofactors in this pathway, or from a genetic abnormality in Hcy metabolism [1]. Both clinical and experimental studies in the last few decades strongly point out that HHcy is an independent risk factor for several pathologies like cardiovascular diseases, neurological and neuropsychiatric disorders, endothelial dysfunction, and kidney diseases [2,3,4]. It is also reported that patients with severe HHcy frequently manifest cardiovascular complications that lead to their mortality [5,6]. In this line, the majority of the experimental studies have indicated cytotoxic damage of vascular endothelium in severe HHcy. Several previous studies have shown that high levels of Hcy can cause endothelial dysfunction through induction of apoptosis in Human Umbilical Vein Endothelial Cells (HUVEC) [7,8]. Increased level of oxidative stress is mostly reported as the main cause of this cellular toxicity and mitochondrial damage and dysfunction as a source of Reactive Oxygen Species (ROS) generation and consequent oxidative damage is the most accepted model in this context [9,10]. However, there are some opposing reports on HHcy-induced higher ROS generation [11] and HUVEC cell viability upon Hcy treatment [12]. Nevertheless, how HHcy induces mitochondrial dysfunction in endothelial cells is not fully understood. ER stress is yet another mechanism that has been repeatedly linked to severe HHcy associated vascular abnormalities, mostly in context of its role in inducing apoptotic cell death [13,14] and inflammation [15,16], but there is very little knowledge regarding the other functional consequences of ER stress in endothelial cells. In general, at population level, moderate HHcy is frequently observed due to aging and nutritional deficiency and a large number of epidemiological and clinical studies have provided compelling evidence that mild to moderate HHcy is associated with increased risk of developing several common vascular diseases including atherosclerosis, stroke, and thrombosis [2,17,18]. However, there are a lack of studies on how moderate HHcy affects endothelial function where, unlike severe HHcy, no apparent cell death is observed.

One of the most important characteristic functional features of endothelial cells is angiogenesis and this process is primarily controlled by the canonical VEGF/VEGFR signaling [19]. At the cellular level, the angiogenic process relies on two key cellular features, endothelial migration and proliferation. Previous reports have shown that severe HHcy inhibits angiogenesis, both in vitro and in vivo [20,21]. Moreover, impairment of endothelial migration and proliferation has also been reported during HHcy [22,23,24]. Though, compared with the consistent observation of HHcy-induced migration defect, a study by Saha et al. reported that endothelial proliferation is not perturbed significantly upon Hcy treatment [25]. Regardless of this controversy, the exact mechanism by which HHcy causes endothelial migration and proliferation defect is not clear. There are also disagreements regarding the association of canonical VEGF/VEGFR signaling, a key regulator of endothelial migration and proliferation, with HHcy. While some studies have indicated it is reduced [26,27], others have suggested it is increased [28,29] during HHcy. Additionally, how endothelial functional impairment like migration and proliferation defects are related to cytotoxicity, particularly in presence of moderate HHcy, remains largely unknown. Another peculiarity of the endothelial cells is their metabolic dependence on glycolysis. Even being highly glycolytic, in response to stress, endothelial cells can undergo metabolic maladaptation, which may act as a major contributing factor to vascular diseases [30,31]. In fact, widely established cardiovascular risk factors like hypercholesterolemia and diabetes are known to cause endothelial dysfunction through metabolic reprogramming [32]. Despite being a metabolic disorder, how HHcy influences metabolism of endothelial cells are less studied. In support of this possibility, a recent study revealed that HHcy accelerates metabolic reprogramming of activated B cells through the glycolytic protein PKM2 [33]. Recently, endothelial metabolism has also gained special attention in respect to the endothelial proliferation [34,35] and that indicates the need for further studies on metabolic changes in Hyperhomocysteinemic endothelial cells.

As the mechanism of endothelial dysfunction in HHcy is unclear, and the prevalence of mild to moderate HHcy is more frequent in population than the rare clinical condition of severe HHcy [36], here we studied the effect of sub-lethal HHcy on the functioning of endothelial cells. Using Hyperhomocysteinemic primary and immortalized HUVEC along with an in vivo zebrafish model we show that a moderate elevation of Hcy induces endothelial dysfunction independent of VEGF/VEGFR suppression at RNA levels and excess ROS production. We demonstrate that two independent axes control Hcy-induced endothelial migration and proliferation. Moderate HHcy causes endothelial migration defect by activating ER stress associated adaptive UPR that leads to abnormal actin cytoskeleton remodeling and ER stress blockers can rescue that migratory defect. However, sub-lethal HHcy induces impairment of endothelial proliferation through defective mitochondrial respiration and ATP production, independent of the ER stress. Through metabolomics and metabolic flux analysis, we show an increase in both TCA cycle and glycolysis in moderate HHcy. We also demonstrate that Hcy-induced mitochondrial respiration-linked ATP loss was a result of dysfunctional ETC in endothelial cells. By reanalyzing a previously published microarray data of moderate HHcy, we finally show that the mechanistic features of endothelial dysfunction in HUVECs are also conserved in adult Hyperhomocysteinemic endothelium which further strengthen our experimental results.

## 2. Materials and Methods

### 2.1. Cell Culture and Chemical Treatments

Immortalized human umbilical vein endothelial cell line (HUVEC/TERT2) was purchased from ATCC (CRL4053, Rockville, MD, USA) and cultured in vascular cell basal medium (ATCC, PCS100030, USA) which was supplemented with endothelial cell growth kit-BBE (ATCC, PCS100040, USA). Primary human umbilical vein endothelial cell (HUVEC) was purchased from Thermo Fisher Scientific (C0035C, Waltham, MA, USA) and grown in M200 medium (Thermo Fisher Scientific, M200500, Waltham, MA, USA) containing large vessel endothelial supplement (LVES) (Thermo Fisher Scientific, A1460801, Waltham, MA, USA). For transduction, Lenti-X HEK 293T cells (Clontech, 632180, CA, USA) were cultured using DMEM high glucose medium (Gibco, USA) which was supplemented with 10% FBS and 1X Antibiotic-Antimycotic. All the cells were maintained in a humidified incubation chamber with 5% CO_2_ at 37 °C. Cells were treated with freshly prepared and filter sterilized Hcy (Sigma-Aldrich, H4628, USA), Cys (Sigma-Aldrich, C9768, St. Louis, MO, USA), 4-PBA (Sigma-Aldrich, P21005, USA) and TUDCA (Selleck Chemicals, S3654, Houston, TX, USA) at indicated doses. For rescue experiments, 4-PBA and TUDCA pretreatments were performed for 2 h and 14 h, respectively.

### 2.2. Zebrafish Maintenance and Morpholino Microinjection

For all the in vivo experiments, adult wild type zebrafish (*Danio rerio*) line ASWT were used, unless otherwise stated. The double transgenic zebrafish line Tg (fli1a:EGFP; gata1a:dsRed) was a kind gift from Dr. Sridhar Sivasubbu’s lab [37]. All the animals were bred, raised, and maintained at 28.5 °C on a 14 h light/10 h dark cycle and were housed in CSIR—Institute of Genomics and Integrative Biology (IGIB), New Delhi, India. All the experiments were performed in compliance with the guidelines laid down by the Institutional Animal Ethics Committee. Care was taken to inflict minimal distress on the animals. Anti-sense morpholino oligonucleotides (MO) were designed and procured from Gene Tools (Philomath, OR, USA). Cocktail of translation blocking oligos against the two CBS isoforms of zebrafish—CBSa and CBSb were used along with a 5-base mismatch specificity control (SC MO). For knockdown of CGL also, a translation blocking morpholino (CGL MO) was used in addition to a 5-base mismatch specificity control (SC MO). Microinjection of MOs were performed following our previously published protocol [38]. Injection replicates with at least 50 percent survival at 24 hpf (hours post-fertilization) were considered for experiments. The sequences of the MOs used are as follows:

CBSa MO: 5′-GGGACTGAAGGCATTATTCCTCAAT-3′
CBSb MO: 5′-CTGGCATGGTTTACCCTGACTATCA-3′
CBS SC MO: 5′-GGCACTCAAGGCAATATACGTCAAT-3′
CGL MO: 5′-CTGCATCTCTGAAGACATGAGTGC-3′
CGL SC MO: 5′-TATGCATATCTGAACACATCAGTCC-3′

### 2.3. Lysate Preparation

For lysis of cell samples, RIPA buffer supplemented with protease inhibitor cocktail (50×) and PhosStop (phosphatase inhibitor; 10×) was used. Lysis of the cells was performed by incubating at 4 °C in the lysis buffer for 30 min with slow rotation. Following incubation, the crude lysate was centrifuged at 10,000 rpm for 20 min at 4 °C. After centrifugation, the supernatant was used as lysate. For zebrafish samples, an equal number of embryos were taken and then subjected to the same above-mentioned protocol except that embryos were homogenized in the NP-40 lysis buffer for about 10–20 s at 800 rpm followed by incubation for 30 min at 4 °C.

### 2.4. Western Blotting

Each sample was mixed with 2× Laemmli buffer and boiled at 95 °C for 5 min. Equal amounts of protein (25–30 μg) for each sample were then loaded on the SDS-PAGE gel of appropriate percentage (10–15%). After resolving, proteins were transferred from gel on PVDF membrane (0.2 μm) using wet transfer method at 4 °C. Membranes were then blocked in 5% skimmed milk or 5% BSA (in case of IRE1p) either for 2 h at room temperature (RT) or overnight at 4 °C. Following blocking, membranes were incubated with the following primary antibodies overnight: anti-GPX1 (Abcam, ab22604, London, UK), anti-SOD1 (Abcam, ab16831, UK), anti-CBS (Abcam, ab96252, UK), anti-CGL (St. John’s Laboratory, STJ27874, London, UK), Anti-GRP78 (BD, 610979, Cell Signalling, Danvers, MA, USA), Anti-IRE1p (Novus, NB1002323, Cell Signalling, Danvers, MA, USA), Anti-ATF4 (CST, 11815, Cell Signalling, Danvers, MA, USA), Anti-CHOP (CST, 2895, Cell Signalling, Danvers, MA, USA), and Anti-Actin (BD, 612657, Becton, San Jose, CA, USA). Membranes were washed three times in 1× TBST (0.1% Tween) for 15 min. Post-washing, HRP-conjugated anti-rabbit or anti-mouse secondary antibodies (Amersham/GE, Buckinghamshire, UK) were added to the membranes and incubated for 2 h. After that, membranes were again washed thrice with 1× TBST (0.1% Tween) for 15 min. Blots were developed by ECL chemiluminescent western blotting substrate using luminol and peroxide and the signals were captured in Syngene^TM^ gel doc system (G:Box). Analysis of the protein bands to quantify fold change was performed using ImageJ software version 1.53t (NIH, Bethesda, MD, USA).

### 2.5. RNA Extraction and qPCR

For cells, RNA was extracted directly using the RNeasy mini kit as per manufacturer’s protocol. For zebrafish, an equal number of embryos (25–30) were first homogenized in 1 mL Trizol^TM^. After homogenization, 200 μL of chloroform was added to the samples followed by centrifugation at 10,000 rpm for 15 min and carefully only the aqueous phase is taken for the next step. The aqueous phase was mixed with 500 μL of absolute ethanol and applied to the columns provided in the RNeasy mini kit (Qiagen, Hilden, Germany). Thereafter, all the subsequent steps were followed as per manufacturer’s instructions in the kit. An equal amount of RNA (1 μg) was used to prepare the cDNA using Verso^TM^ cDNA synthesis kit. qPCR run was carried out on a LightCycler 480 system (Roche, Basel, Switzerland). All experiments were performed in a 384-well plate with triplicates of each sample. The details of the primers used are given in Table S1. Results were normalized with 18S.

### 2.6. Homocysteine Measurement

Intracellular Hcy concentration was measured by HPLC. Samples were lysed using RIPA buffer. After that, 100 μL of the lysate was taken and then treated with 35 μL of 1.43 M sodium borohydride in 0.1 M NaOH (for reduction in disulfide bonds). Next, in order to avoid foaming, 10 μL of amyl alcohol was added. Thereafter, 35 μL of 1 M HCl was added. To this mixture, 50 μL of 7 mM Bromobimane in 5 mM sodium EDTA (pH 7.0) was added (for conjugation of the reduced thiols with fluorophore) and the solution was then incubated at 4 °C for around 15 min followed by incubation at RT for another 30–45 min. For precipitation of intracellular proteins, 50 μL of 1.5 M perchloric acid was used followed by centrifugation at 12,000 rpm for 10 min. Then, neutralization of the supernatant was performed with 6 μL of 2 M Tris base. For injection, 4 μL of derivatized sample was then used and this was loaded into a 100 × 4.6 mm, 1.8 micron Eclipse plus C18 column using Agilent-1260 Ultra-high performance liquid chromatography (UPLC). Equilibration of the Column was performed with 90% buffer A (composed of 5% methanol and 0.86% acetic acid in water) and 10% Buffer B (100% methanol). Lastly, elution of thiols was performed by linear gradient of both buffers (from 90% Buffer A, 10% Buffer B to 0% Buffer A and 100% buffer B in 7 min). A flow rate of 0.7 mL/min was maintained at RT. A reference standard curve was generated by using the known concentrations of Hcy. Final estimation of Hcy level in the samples were performed by measuring the area for the homocysteine-bimane peak followed by calculation of its concentration using a regression equation derived from the standard curve.

### 2.7. Trypan Blue Exclusion Assay

Endothelial cells at a density of 8 × 10^4^ were seeded to each well of a 12-well flat bottom culture plate. Following treatment, live as well as dead cells (floaters) were collected and then centrifugation was performed at 3000 rpm for 5 min. After that, the supernatant was discarded, and cells were dissolved in 1× PBS. For staining, 10 μL of the cell suspension was mixed with 0.4% trypan blue solution (Gibco) and then incubated for 2 min at RT. Live cells (trypan negative) and dead cells (trypan positive) were counted with the help of a hemocytometer and cell death was represented in percentage of dead cells.

### 2.8. Tube Formation Assay

Control and 2 mM Hcy treated (24 h) HUVEC/TERT2 cells were used for the tube formation assay. Firstly, wells of a 48-well plate were coated with 125 μL of Matrigel^TM^ (Corning 356234) matrix by adding the Matrigel^TM^ towards the center of the well and then spreading with a tip. During coating, Matrigel^TM^ was kept on ice and pre-chilled 48-plate and tips were used. While adding Matrigel^TM^ to the wells, special care was taken to avoid creating any bubbles by not squeezing the pipette till the very last stop. The plates were then kept at 37 °C for 1 h. After coating, 150 μL of untreated or Hcy treated (2 mM for 24 h) cell suspension (40,000 cells/well) in complete growth medium was added to each well. Lastly, the plates were incubated (without Hcy) at 37 °C for 3–4 h followed by bright field imaging at 4× magnification. Measurement of total tube length and number of branches were performed for three different fields in each well using the Angiogenesis Analyzer plugin in ImageJ [39].

### 2.9. Scratch Wound Assay

Cells were plated in a 12-well plate (1 × 10^5^) and cultured until 100% confluency. After that, cells were starved in incomplete growth medium (without serum and growth factors) for 8 h. Following starvation, manually two straight line scratches were created diametrically perpendicular to each other through the center of the plate with a P200 pipette tip and the cells were allowed to migrate for 24 h in presence of Hcy and/or other chemicals (Cys, 4-PBA, TUDCA). To exclude cellular proliferation-induced changes, Mitomycin C (Sigma-Aldrich, USA) was added to each well at a final concentration of 1 μg/mL. Just before imaging, scratches for 0 h time points were made in separate wells of the same plate. Cells were then fixed by adding a fixing solution (37% formaldehyde and 0.2% Glutaraldehyde in PBS) followed by 10 min of incubation at RT and finally bright field images were obtained at 10× magnification. Migrated cells were counted from 6 different image fields from each well using ImageJ software version 1.53t (NIH, USA) and the difference was reported as fold change in migrated cells.

### 2.10. BrdU Incorporation Assay

Proliferation of cultured human endothelial cells was estimated by BrdU Cell Proliferation ELISA kit (Roche), following manufacturer’s protocol. Briefly, 1 × 10^4^ cells were seeded in a 96-well flat bottom plate containing 100 μL media in each well. After that, BrdU labeling solution was added to the cells (final concentration: 10 μM. Per well). The plate was then incubated for 3 h in a cell culture incubator at 37 °C. The BrdU containing media was then removed and cells were fixed and denatured by adding FixDenat for 30 min at RT. After removing FixDenat, cells were incubated with 100 μL of anti-BrdU-peroxidase (POD) working solution for 90 min at RT. Antibody conjugate was then removed and the cells were washed thrice with 200 μL of 1× PBS. Finally, to each well, 100 μL of substrate solution was added and after 2–5 min the absorbance was measured in a microplate reader at 370 nm taking 492 nm as reference wavelength.

### 2.11. Measurement of Mitochondrial Membrane Potential

Mitochondrial membrane potential was quantified using a potentiometric dye called Tetramethylrhodamine ethyl ester or TMRE (Invitrogen, Life technologies, Waltham, MA, USA). Cells were seeded at 1 × 10^4^ density in a black 96-well plate with flat clear bottom. After Hcy treatment, TMRE was added to each well at a final concentration of 400 nM and then the plate was incubated for 10 min in the dark at 37 °C. As a positive control, prior to TMRE addition, cells were incubated with an uncoupler called carbonyl cyanide m-chlorophenyl hydrazone or CCCP (Invitrogen) at final concentration of 500 μM for 10 min at 37 °C in the dark. Fluorescent intensities were measured in a microplate reader with 549 nm and 575 nm as excitation and emission wavelengths, respectively.

### 2.12. Measurement of Cellular Reactive Oxygen Species (ROS)

Total cellular ROS in HUVEC/TERT2 cells was analyzed using the fluorescent dye 2′, 7′- dichlorodihydrofluorescein diacetate (CM-H2DCFDA, Invitrogen). Briefly, 1 × 10^4^ cells were seeded in a black 96-well plate with flat clear bottom. The next day, cells were treated with Hcy for 24 h. Post-Hcy treatment, CM-H2DCFDA was added to each well at a final concentration of 25 μM followed by incubation at 37 °C for 15 min in the dark. Before CM-H2DCFDA addition, positive control wells were subjected to 1 mM H_2_O_2_ treatment for 10 min. Mean fluorescent intensities were measured using a microplate reader taking 492 nm as excitation wavelength and 517 nm as emission wavelength.

For zebrafish embryos, 2 dpf embryos were exposed to 5 μM CM-H2DCFDA in embryo water and incubated for 20 min at 28.5 °C. After that, embryos were washed three times with embryo water. Finally general ROS was observed under the green channel of Axioscope A1 (Carl Zeiss, Oberkochen, Germany) fluorescence microscope. For positive control, 1 mM H_2_O_2_ was used.

### 2.13. Generation of mitoGFP Transduced Cells

For lentivirus transduction, pLYS1-FLAG-MitoGFP-HA (Addgene 50057) plasmid was used. Transfection of the cells and collection of lentiviral particles were performed as per our previously published protocol [40].

### 2.14. Confocal Imaging of mitoGFP Transduced Cells

To check mitochondrial morphology, MitoGFP transduced cells were grown on coverslips in a 6-well plate. Post-Hcy treatment, cells were fixed using the aforementioned fixing solution for 10 min at RT. After a wash with 1× PBS, cells were mounted on the slides using ProLong Gold mounting solution with DAPI (Invitrogen, USA). Images were captured by Nikon confocal A1R HD attached with Ti2-E (Nikon, Tokyo, Japan) using 60× Nikon objective (1.42 NA).

### 2.15. Mitochondrial Length Measurement

Measurement of mitochondrial size in MitoGFP transduced HUVEC/TERT2 cells was performed using ImageJ as per our previously published protocol [41]. Briefly, three channels from the images were separated and then the green channel was selected for (labeled mitochondria) sharpening. After that, despeckle, background subtraction and enhancement of local contrast were performed. Considering mitochondria as a filamentous tube-like structure, a filter called ‘tubeness’ (sigma = 0.0210) was imposed to increase the filamentous feature of segmented mitochondria. After that, gaussian blur (sigma radius = 1.000) was applied before finally converting the image to binary. This image was then used to measure mitochondrial length.

### 2.16. Rhodamine Phalloidin Staining

F-actin staining of endothelial cells was performed using rhodamine labeled phalloidin. At first, HUVEC/TERT2 cells were grown on coverslips in a 6-well plate. After treatment, cells were fixed using previously mentioned fixing solutions for 10 min at RT. Then, one wash with 1× PBS was given. Thereafter, cells were permeabilized in 0.1% Triton X-100 in PBS for 5 min at RT to increase permeability. Samples were then washed twice in 1× PBS. Stock solution (400× in DMSO) of rhodamine phalloidin (Invitrogen) was diluted (2.5 μL in 1 mL of PBS) and then added to the samples followed by incubation in a cell culture incubator at 37 °C for 60 min. Then the samples were washed three times with 1× PBS. Finally, coverslips were mounted using Prolong Gold mounting solution with DAPI. Confocal images were acquired using Nikon confocal A1R HD attached with Ti2-E. Images were captured using a 100× Nikon objective (1.45 NA).

### 2.17. Zebrafish Imaging and Heartbeat Measurement

For imaging, MO injected embryos were immobilized in 2.5% methyl cellulose and bright field images were taken at 48 hpf using Nikon SMZ800N Stereomicroscope at 5× magnification. Fluorescent images were captured in Zeiss Axioscope A1 (Carl Zeiss, Germany) at 5× magnification under GFP channels. To study intersegmental vessels, zebrafish embryos were mounted in low melting point agarose and confocal imaging was performed using Leica SP8 TCS microscope (Leica, Wetzlar, Germany) at 10× magnification. Images were captured and processed with Zeiss AxioVision 4.6 and Leica LAS X software version 5.1, respectively. The heartbeat of the embryos was measured by the stopwatch count method. Analysis of zebrafish vasculature and pericardial area was performed using the ImageJ software version 1.53t (NIH, USA).

### 2.18. Glucose Uptake Assay

After 24 h of Hcy treatment, cells were replenished with HBSS containing 2-NBDG (Invitrogen, USA) of 10 μM final concentration and incubated at 37 °C for 30 min. Fluorescence was measured in a microplate reader with 475 nm and 550 nm as excitation and emission wavelengths, respectively.

### 2.19. Extracellular Flux Analysis

Oxygen consumption rate (OCR) and extracellular acidification rate (ECAR) was measured using Seahorse XF24 extracellular flux analyzer (Agilent Technologies, Santa Clara, CA, USA). HUVEC/TERT2 cells were seeded at a density of 8 × 10^4^ per well. As per manufacturer’s protocol, Mitochondrial stress test was performed in basal condition as well as in stressed condition in presence of 1 μM oligomycin, 1 μM FCCP and 0.5 μM rotenone/antimycin A (all from Agilent). Glycolysis stress test was run in basal condition and in presence of 10 mM glucose, 1 μM oligomycin and 25 mM 2-deoxy-glucose or 2-DG (all from Agilent). OCR and ECAR values quantified by these experiments were normalized to total protein content of each sample, calculated by performing BCA assay after each run. For analysis, pre-standardized tools in the Seahorse Wave software version 2.6.3 were used.

### 2.20. Metabolite Extraction and Targeted Metabolomics

Treated and non-treated HUVEC/TERT2 cells were harvested, washed once with ice-cold PBS followed by centrifugation for 5 min at 1500 rpm (in 4 °C). Then, cells were resuspended in pre-chilled methanol:water (80%:20% respectively) solution for approximately 10 min with intermittent vortexing. This suspension was then centrifuged at 10,000 rpm for 15 min in 4 °C. Thereafter, the supernatant was collected and transferred into a fresh microcentrifuge tube and vacuum dried in a vacuum concentrator in alcoholic mode at 30 °C. The lyophilized metabolite was then re-suspended in 0.1% formic acid in water, vortexed and centrifuged at 10,000 rpm for 5 min. Sufficient volume of supernatant was then transferred into micro volume inserts and injected into the LC/MS system.

Data acquisition was performed using a Thermo TSQ—Altis triple stage quadrupole mass spectrophotometer in dual polarity (both positive and negative) mode. The instrument voltage was as follows: capillary voltage = 3500 V and 3000 V for positive and negative mode respectively. Onto a UPLC BEH HILIC column (Waters Corp., Milford, MA, USA), 10 μL of sample was loaded. Metabolites were eluted from the column at a flow rate of 0.300 mL/min. The mobile phase consisted of a linear gradient of 100% LC grade water with 0.1% formic acid (Buffer A) and 95% Acetonitrile with 0.1% formic acid (Buffer B). Relative quantification was performed using Skyline software (version 1.2.2). The optimized parameters of all the different measured metabolites are given in Table S2. Fold change in peak area was calculated and represented as fold change in AUC (area under curve).

### 2.21. Analysis of Available Microarray Dataset

The previously published microarray dataset GSE175735 from Gene Expression Omnibus (GEO) database was used to understand the effect of HHcy on human adult endothelial cell line (HAEC) [42]. Files were extracted and imported into R using the readAffy() function. Samples were pre-processed in order to remove any systematic error such as background noise and dye bias. Pre-processing involves quantile normalization and background correction using rma() function. Then gene probes were annotated with respective Gene IDs by hugene10sttranscriptcluster.db package. Thereafter, differentially expressed genes (DEGs) between Hcy treated (three replicates) and control samples (three replicates) were obtained using the limma package. Genes with *p*-value < 0.05 were considered for DEG analysis. After that, DEGs were mapped to KEGG (Kyoto Encyclopedia of Genes and Genomes) pathways. For this purpose, all the gene entries from Cell Cycle (has04110), Migration (hsa04670), UPR (has04141), Glycolysis (hsa00010), TCA cycle (hsa00020), Angiogenesis (has04370), and Antioxidants (hsa05208) were downloaded. Heatmaps of DEGs were plotted for each pathway using the coolmap() function of the limma package [43].

### 2.22. Statistical Analysis

All the data are from three independent experiments. Quantitative statistical data are reported as means ± SEM. Calculation of statistical significance was performed by unpaired Student’s *t*-test and represented by asterisk with * *p* ≤ 0.05, ** *p* ≤ 0.01, *** *p* ≤ 0.001, **** *p* ≤ 0.0001 and ns as non-significant (*p* > 0.05). All the graphs were made using GraphPad Prism (GraphPad Software version 9, La Jolla, CA, USA).

## 3. Results

### 3.1. Moderate Increase in Hcy Levels Causes Endothelial Dysfunction

**Figure 1:**
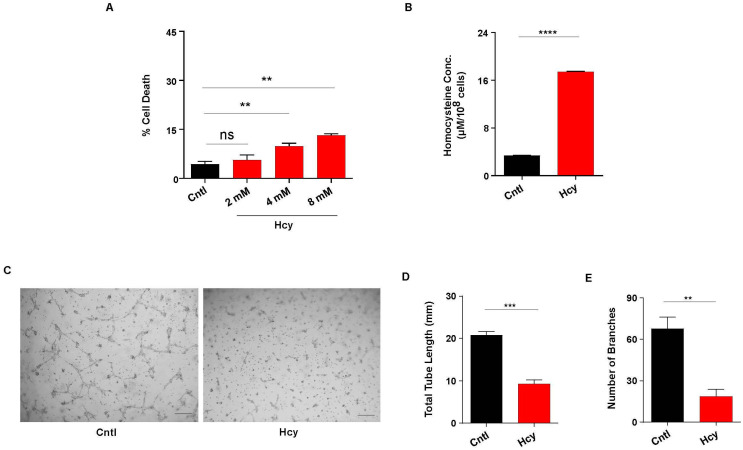
Sub-lethally increased Hcy causes endothelial dysfunction. (**A**) Bar graph represents percentage of cell death in HUVEC/TERT2 cells treated with increasing concentration of Hcy for 24 h. A sub-lethal concentration of 2 mM (24 h) was chosen for all the subsequent experiments. (**B**) HPLC mediated quantification of intracellular Hcy concentration in HUVEC/TERT2 cells revealing induction of moderate Hyperhomocysteinemic condition post 2 mM Hcy treatment for 24 h. (**C**) Representative images of tube formation assay showing functional abnormality in Hcy treated HUVEC/TERT2 cells compared with untreated cells. Scale bar, 250 μm. (**D**,**E**) Respective quantifications showing total tube length and number of branches formed during tube formation assay are drastically reduced upon 2 mM Hcy treatment for 24 h. Data are shown as Mean ± SEM with *n* ≥ 3. ** *p* ≤ 0.01, *** *p* ≤ 0.001, **** *p* ≤ 0.0001 and ns is non-significant (*p* > 0.05).

In order to understand the effect of HHcy on endothelial function, we first exposed immortalized human endothelial cell line HUVEC/TERT2 to various doses of Hcy for 24 h. Cell death analysis by Trypan blue exclusion assay showed that Hcy can cause cytotoxicity in endothelial cells in a dose dependent manner. We found that even at a very high concentration (8 mM for 24 h), the amount of cell death was below 15%, which indicates a moderate impact of Hcy on survival of endothelial cells (Figure 1A). To further negate the effect of cytotoxicity on subtle endothelial function, we chose 2 mM concentration (24 h) for all our subsequent experiments as at this dose no statistically significant cell death was observed. To further check the amount of Hcy actually entered into the cell after 24 h of treatment, we measured the intracellular level of Hcy in treated and non-treated cells by HPLC. Our result showed that the intracellular concentration of Hcy was around 18 μM range upon treatment (Figure 1B), a finding consistent with the previous report [44] and with the moderate HHcy condition. To study the functional consequence of this moderate sub-lethal HHcy on endothelial cells, we performed a tube formation assay. As shown in the representative images, tube formation was drastically hampered upon Hcy treatment (Figure 1C). Quantitative analysis also revealed that in comparison to untreated cells, total tube length and number of branches were reduced by around 50% in Hcy treated cells (Figure 1D,E). Overall, we found that sub-lethal Hcy exposure leading to induction of moderate HHcy can detrimentally influence endothelial function.

### 3.2. Moderate HHcy Impairs Endothelial Migration and Proliferation without Suppressing the Expression of VEGF/VEGFR Transcripts and ROS Level Change

**Figure 2:**
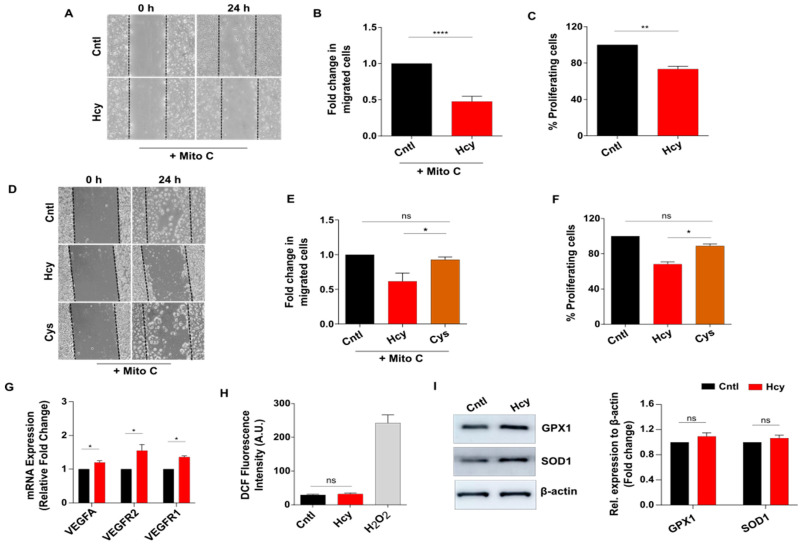
Sub-lethal HHcy reduces endothelial migration and proliferation without suppressing VEGF/VEGFR transcripts and ROS level change. (**A**) Scratch wound assay in presence of Mitomycin C showing less migrated endothelial cells at 24 h post 2 mM Hcy treatment. (**B**) Quantification of migrated cells revealing that endothelial migration is significantly reduced in Hcy treated cells compared with control cells. (**C**) Bar plot of BrdU cell proliferation assay indicating that 2 mM Hcy treatment for 24 h causes proliferation defect in endothelial cells. (**D**) Representing scratch wound assay images depicting that as opposed to Hcy treatment, a similar concentration of Cys did not affect migration of endothelial cells when compared with untreated cells at 24 h. (**E**) Bar plot of measurement of migrated cells in scratch wound assay showing that contrary to Hcy treated cells, fold change in migrated cells is not altered upon 2 mM Cys treatment for 24 h compared with control cells. (**F**) BrdU cell proliferation assay revealing that unlike Hcy treated cells, 2 mM Cys treatment for 24 h did not influence endothelial proliferation. (**G**) Bars showing that compared with untreated cells, exposure to sub-lethal Hcy caused upregulation of mRNA levels of canonical VEGF signaling markers. 18S was used as internal control. (**H**) Bar graph showing that sub-lethal Hcy treatment does not induce ROS production in endothelial cells as determined by the fluorescent probe CM-H2DCFDA. For positive control, H_2_O_2_ was used. (**I**) Representative western blots showing protein levels of major antioxidant markers GPX1 and SOD1. Corresponding bar graphs showing densitometric analysis of the protein bands which suggest a non-significant but slight trend of upregulation of both the proteins in 2 mM Hcy (24 h) treated cells. As a loading control β-actin was used. Data are shown as Mean ± SEM with *n* ≥ 3. * *p* ≤ 0.05, ** *p* ≤ 0.01, **** *p* ≤ 0.0001 and ns is non-significant (*p* > 0.05).

Next, we wanted to investigate how a sub-lethal dose of Hcy affects migration and proliferation, two critical parameters of endothelial function. Scratch wound assay in presence of proliferation blocker Mitomycin C revealed that at a sub-lethal concentration, Hcy can significantly impair endothelial migration (Figure 2A,B). Moreover, BrdU incorporation assay showed that endothelial proliferation is also inhibited by around 20% upon Hcy treatment (Figure 2C). To check whether the above-mentioned effects of sub-lethal HHcy are conserved in a more physiological cellular set-up of primary endothelial cells, we used primary HUVEC. We confirmed that the treatment of primary HUVEC with 2 mM concentration of Hcy for 24 h faithfully recapitulated the defects in migration and proliferation at the absence of cellular toxicity, as observed in HUVEC/TERT2 cells (Figure S1A–C). These results demonstrated that the functional response of HUVEC/TERT2 towards exogenous Hcy was comparable to that of primary endothelial cells.

Then we tested whether the observed functional abnormalities were specific to Hcy and not a general response of endothelial cells toward any high sulfhydryl group containing metabolite. To verify this, we exposed HUVEC/TERT2 cells to 2 mM of Cysteine, another sulfhydryl group containing amino acid from the same metabolic pathway, for 24 h. Through scratch wound assay we observed that in contrast to Hcy, treatment with Cysteine did not alter migration of endothelial cells (Figure 2D,E). Similarly, BrdU incorporation assay showed that unlike Hcy, proliferation of endothelial cells also did not change significantly upon Cysteine exposure (Figure 2F). Since a number of previous studies have implicated canonical VEGF/VEGFR signaling, a master regulator of endothelial migration and proliferation, with HHcy-associated vascular dysfunction [26,28], we assessed the expression levels of VEGFA ligand and its cognate receptors VEGFR1 and VEGFR2. As shown by qPCR data, sub-lethal Hcy treatment did not cause suppression of VEGFA, VEGFR1 and VEGFR2 at transcript levels (Figure 2G). Another mechanistic aspect that has been most frequently linked with HHcy-induced vascular endothelial dysfunction is oxidative stress [9,10]. Intriguingly, total ROS measurement using general ROS indicator CM-H2DCFDA showed that 2 mM Hcy treatment did not cause an increased ROS level in endothelial cells (Figure 2H). We further checked the expression of two major antioxidant proteins in Hcy-treated cells. Our data showed that there were no statistically significant changes in the expression of GPX1 and SOD1 upon treatment with 2 mM Hcy for 24 h, though we saw an increasing trend in their expression (Figure 2I). As a whole, these data indicate that sub-lethal HHcy can specifically impede endothelial migration and proliferation, which is not directly related to the lowering of canonical VEGF/VEGFR transcripts and increased ROS production.

### 3.3. Generation of In Vivo Knockdown Models of CBS and CGL, Transsulfuration Pathway Regulators Involved in Hcy Catabolism

**Figure 3:**
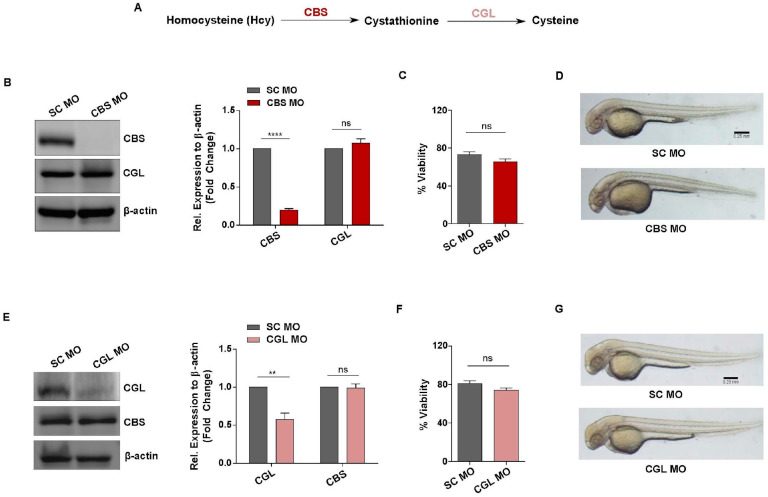
Generation of in vivo knockdown models of CBS and CGL, regulators of transsulfuration pathway involved in Hcy catabolism. (**A**) Simplified diagram showing the enzymes and metabolites of transsulfuration pathway (**B**) Representative western blot of 2 dpf embryos lysates showing that compared with SC MO injected embryos, CBS protein level is downregulated in CBS MO injected embryos. Expression of CGL, the other enzyme of the same pathway, remained unaltered. As a loading control β-actin was used. Corresponding bar plot showing fold change in protein expression (normalized to β-actin) as determined by densitometric analysis of the protein band. (**C**) Bar plot revealing no statistically significant difference in the viability of scrambled control and Hyperhomocysteinemic CBS MO embryos. (**D**) Bright field images exhibiting no apparent gross morphological defect in CBS morphants compared with scrambled Mo injected embryos of 2 dpf. Scale bar, 0.25 mm. (**E**) Representative western blot of embryo lysates at 2 dpf showing reduced protein level of CGL in the embryos injected with CGL MO. Expression of CBS, the upstream protein of the same pathway, remained unaltered. β-actin was used as a loading control. Corresponding bar diagram showing densitometric analysis of the fold change in protein expression (normalized to β-actin). (**F**) In comparison to SC MO injected embryos, viability of CGL MO injected embryos were not altered significantly. (**G**) Representative bright field images showing absence of any gross morphological defect in CGL MO injected embryos compared with SC MO injected ones at 2 dpf. Scale bar, 0.25 mm. Data are shown as Mean ± SEM with *n* ≥ 3. ** *p* ≤ 0.01, **** *p* ≤ 0.0001 and ns is non-significant (*p* > 0.05).

To examine whether the observed functional implications of sub-lethal HHcy on vascular endothelial cells are conserved in vivo, we used zebrafish as a model organism. For this purpose, we targeted CBS, the rate limiting enzyme of the transsulfuration pathway that catalyzes the conversion of Hcy to cystathionine [45] (Figure 3A). As our goal was to study the effect of moderate HHcy, we used a genetic knockdown (KD) approach in our model generation instead of a CBS knockout (KO) model which mimic Homocystinuria, a severe form of HHcy [6,46]. To generate moderate HHcy in zebrafish, we used a cocktail of translation blocking morpholino oligonucleotides (MOs) targeting the ATG regions of two zebrafish paralogs CBSa and CBSb (referred to as CBS MO hereafter). We also incorporated a 5-base mismatched specificity control MO (SC MO) to generate control embryos. For translational knockdown, MOs were microinjected at one cell stage and then embryos were observed at 2 days post-fertilization (dpf) as by this time most of the components of the cardiovascular system have already developed. At first, we confirmed the efficacy of the MOs through western blotting which revealed that CBS is significantly downregulated in CBS MO injected embryos (morphants) compared with SC MO injected embryos. We also checked the protein level of CGL, the downstream gene of CBS, as a negative control and found that CGL level is not altered in CBS MO injected embryos (Figure 3B). Moreover, HPLC mediated measurement from our lab previously established that knockdown of CBS in zebrafish embryos can cause 4–5-fold increase in Hcy levels representing moderate HHcy [47]. Next, we checked the viability of CBS MO and SC MO injected embryos and found that moderate HHcy is not lethal in vivo which nicely corroborates with our cellular model data (Figure 3C). Additionally, compared with the anatomy of SC MO injected embryos, sub-lethal HHcy did not cause any apparent gross morphological defect in CBS MO injected embryos (Figure 3D).

The transsulfuration pathway acts as the major intracellular source of Cysteine and the first enzymatic step of this pathway is governed by CBS. Thus, we wondered whether general perturbation of the transsulfuration pathway would mimic the effect of HHcy on the vasculature in vivo. To check that, we also generated an in vivo zebrafish model of Cystathioninuria, a clinical condition associated with deficiency of CGL [48], the second enzyme of transsulfuration pathway [45] (Figure 3A). Similar to generation of Hyperhomocysteinemic embryos, a translation blocking MO against the ATG start site of CGL was used along with a 5-base mismatch specificity control. At first, western blotting of the embryo lysates confirmed that CGL is significantly downregulated in the morphant embryos. Although CBS, which is the upstream gene of the pathway, remained unaltered in the morphant embryos suggesting the morpholino is specific and functional (Figure 3E). As is the case with HHcy, viability of the morphant embryos did not decline significantly (Figure 3F) and there were no obvious gross morphological defects in CGL deficient embryos (Figure 3G). Thus, we generated in vivo models to study the response of vasculature towards sub-lethal HHcy as well as a disturbed transsulfuration pathway per se.

### 3.4. Moderate HHcy Induces Vascular Abnormality In Vivo without Suppressing the Expression of VEGF/VEGFR Transcripts and ROS Level Change

**Figure 4:**
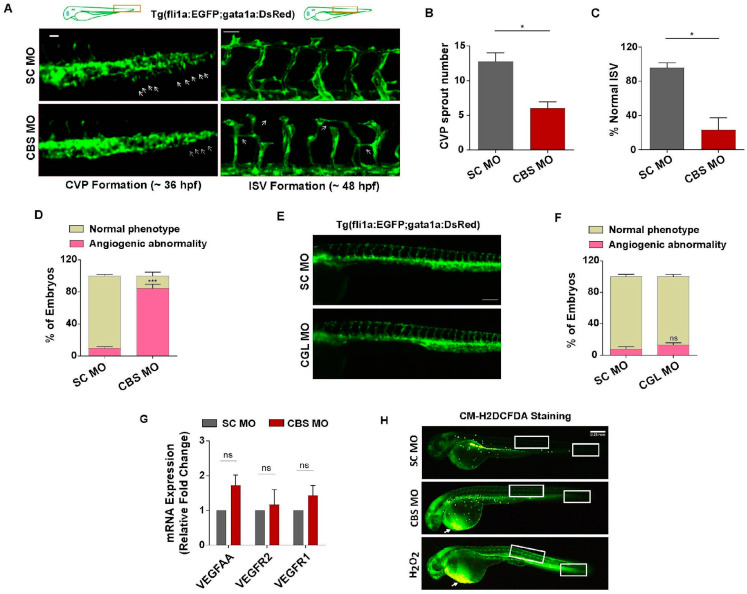
Sub-lethal HHcy causes vascular abnormality in vivo without suppressing VEGF/VEGFR transcripts and ROS level change. (**A**) Upper panel: schematic illustration of transgenic zebrafish line Tg(fli1a:EGFP;gata1a:DsRed) showing anatomical position (rectangle) of CVP and ISV forming area in 36 hpf and 48 hpf embryos respectively. Left panel: representative images revealing impairment in sprouting angiogenesis during CVP formation of CBS morphants. Arrows are depicting CVP sprouts. Scale bar, 0.05 mm. Right panel: representative confocal images showing abnormal ISV formation in CBS morphants compared with SC MO injected embryos. Arrows indicating incomplete, irregular, and abnormally branched ISVs. Scale bar, 50 μm. (**B**,**C**) Bar plots respectively showing CVP sprout number and percentage of normal ISV are significantly reduced in CBS morphants. (**D**) Bar diagram depicting percentage of embryos with angiogenic abnormality is significantly higher in CBS MO injected embryos compared with SC MO injected ones. (**E**) Representative fluorescent images showing no observable difference in the vascular anatomy of SC MO and CGL MO injected embryos at 2 dpf. Scale bar, 0.2 mm. (**F**) Bar plot depicting no significant change in percentage of embryos with observable angiogenic abnormality in CGL morphants compared with SC MO injected ones. (**G**) Bars showing mRNA expression levels of canonical VEGF signaling markers in CBS MO and SC MO injected embryos at 2 dpf. 18S was used as internal control. (**H**) Representative images depicting location of oxidative stress in 2 dpf zebrafish embryos, detected using general ROS indicator CM-H2DCFDA. White arrows showing drastic increase in ROS levels in the pericardial area of CBS morphants and H_2_O_2_ treated embryos. White boxes of the tail and regions representing sites of previously observed CVP and ISV angiogenesis, respectively. Scale bar, 0.25 mm. Data are shown as Mean ± SEM with *n* ≥ 3. * *p* ≤ 0.05, *** *p* ≤ 0.001 and ns is non-significant (*p* > 0.05).

In order to assess vascular endothelial function upon induction of sub-lethal HHcy in zebrafish embryos, we used transgenic zebrafish line Tg(fli1a:EGFP;gata1a:DsRed) where endothelial cells express EGFP under the fli1a promoter and analyzed caudal vein plexus (CVP) and intersegmental vessel (ISV) formation, two angiogenic events of distinct anatomical regions occurring at different time points [49]. Compared with the normal sprouting angiogenesis of SC MO injected embryos, we observed that formation of CVP sprouts is drastically reduced in CBS MO injected embryos (Figure 4A, left panel). Upon quantification we found a 50% decrease in the number of CVP sprouts of the morphant embryos (Figure 4B). Likewise, in comparison to SC MO injected embryos, the angiogenic sprouts associated with ISV formation appeared to be irregular, incomplete, and abnormally branched in the morphant embryos (Figure 4A, right panel). The percentage of embryos with normally formed ISV also found to be severely reduced in CBS MO injected embryos (Figure 4C). Furthermore, phenotypic screening revealed that around 80% of embryos with sub-lethal HHcy exhibited angiogenic abnormalities (Figure 4D). Interestingly, apart from vascular dysfunction, the morphant embryos later (4 dpf) developed severe pericardial edema characterized by a tubular heart (Figure S2A) and significant increase in the pericardial area (Figure S2B). Along with cardiac structural anomaly, we also observed functional impairment in terms of reduction in the heartbeat of morphant embryos compared with SC MO injected ones (Figure S2C). As opposed to Hyperhomocysteinemic embryos, CGL deficient embryos showed no evident anatomical differences in the vasculature compared with control embryos (Figure 4E). The percentage of embryos with angiogenic abnormalities were also not significantly higher in CGL morphants (Figure 4F). Therefore, we found that perturbation of the transsulfuration pathway cannot by itself cause vascular dysfunction in vivo. Nevertheless, similar to our cellular model, we also checked the transcriptional status of canonical VEGF signaling markers in the embryos with sub-lethal HHcy and found that mRNA levels of VEGFAA, VEGFR2 and VEGFR1 are not reduced (Figure 4G). Furthermore, the general ROS indicator CM-H2DCFDA-mediated staining revealed that although there is a sharp increase in ROS levels of the pericardial region, CVP and ISV forming regions of hyperhomocysteinemic embryos have no such striking changes. In contrast, H_2_O_2_ treated positive control embryos have increased ROS production not only in the pericardial region but also in the tail and trunk, CVP and ISV forming anatomical parts respectively (Figure 4H). As a whole, these results suggest that sub-lethal HHcy can specifically cause vascular endothelial dysfunction in vivo and as is the case with our cellular model data, it is not associated with inhibition of VEGF/VEGFR at RNA levels and ROS induction.

### 3.5. Adaptive UPR Activation Controls Endothelial Migration Defect in Moderate HHcy

**Figure 5:**
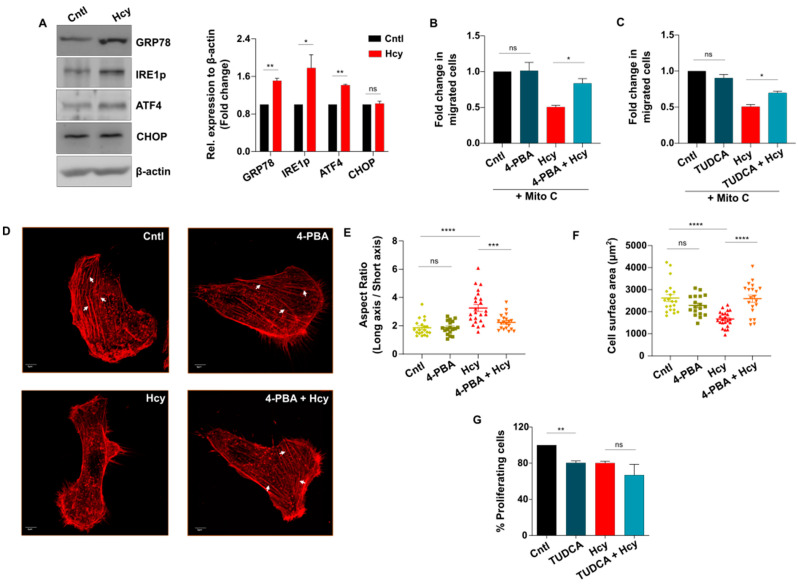
Sub-lethal HHcy-induced adaptive UPR controls endothelial migration defect. (**A**) Representative western blots showing protein levels of UPR markers GRP78, IRE1p and ATF4 are upregulated upon 2 mM Hcy treatment for 24 h. Terminal UPR marker CHOP remained unaltered post-sub-lethal Hcy treatment. As a loading control β-actin was used. Corresponding bar graph showing densitometric analysis (normalized to β-actin) of the blots. (**B**,**C**) Respective quantifications of scratch wound assay at 24 h revealing that chemical chaperone 4-PBA (1 mM) and TUDCA (1 mM) can significantly improve sub-lethal HHcy-induced endothelial migration defect. (**D**) Representative confocal images of rhodamine-phalloidin stained endothelial cells showing that sub-lethal Hcy-induced abnormally elongated cell morphology as well as actin stress fiber (white arrows) disappearance are rescued by 4-PBA pre-treatment. Scale bar, 5 μm. (**E**) ImageJ based analysis demonstrating that 4-PBA pre-treatment reversed the aberrant reduction in cellular aspect ratio (major axis/minor axis), induced by 24 h treatment of 2 mM Hcy. (**F**) Quantification by ImageJ suggesting that exposure to sub-lethal Hcy significantly decreased the surface area of endothelial cells which was rescued by 4-PBA. (**G**) Bar plot showing no beneficial effect of chemical chaperone TUDCA on impairment of endothelial proliferation caused by sub-lethal Hcy treatment. Data are shown as Mean ± SEM with *n* ≥ 3. * *p* ≤ 0.05, ** *p* ≤ 0.01, *** *p* ≤ 0.001, **** *p* ≤ 0.0001, and ns is non-significant (*p* > 0.05).

Since we found that sub-lethal HHcy invokes a similar functional abnormality in vitro and in vivo, which is independent of suppressed expression of VEGF/VEGFR transcripts and ROS induction, we next focused to elucidate the mechanism by which migration and proliferation is impaired in Hyperhomocysteinemic endothelial cells. In the context of Hcy-induced cytotoxicity, generation of ER stress is a common mechanism that has been frequently linked in both endothelial [13,50,51] and other cell types [52,53]. As a response to ER stress, the unfolded protein response (UPR) pathway is known to be activated in all cells [54]. Therefore, we investigated whether ER stress and concomitant UPR activation plays any role in sub-lethal HHcy-induced endothelial dysfunction as well. Western blot analysis revealed that sub-lethal Hcy treatment causes activation of ER stress-associated UPR as evident by significant induction of three well-known markers, namely, early UPR regulator protein GRP78, UPR transducer phosphorylated IRE1 (IRE1p) and UPR effector protein ATF4. An unresolved or sustained UPR may induce apoptosis majorly through induction of CHOP [55]. Interestingly, we detected no significant change in the expression level of CHOP upon exposure to sub-lethal HHcy (Figure 5A). Thus, we found that sub-lethal HHcy induces ER stress and concurrently activates adaptive UPR which does not lead to endothelial apoptosis. Thereafter, we hypothesized that ER stress-associated adaptive UPR activation is the sole cause of sub-lethal HHcy-induced endothelial migration and proliferation defects. To explore this possibility, we used 4-PBA and TUDCA, two commonly used chemical chaperones with well-known ER stress reducing properties [56]. Firstly, we confirmed alleviation of ER stress by reduction in the aberrant increase in GRP78 in case of both of the chemical chaperones (Figure S3A,B). Subsequently, through scratch wound assay we discovered that pre-treatment with 4-PBA and TUDCA could significantly rescue sub-lethal Hcy-induced impaired endothelial migration (Figure 5B,C and Figure S3C,D). Since migration of endothelial cells involves specific cytoplasmic changes that depend on remodeling of the actin cytoskeleton [57,58], we then carried out rhodamine phalloidin based F-actin staining to understand the basis of migratory abnormality in Hyperhomocysteinemic endothelial cells. As shown in the representative confocal images, we observed that when subjected to 2 mM Hcy treatment for 24 h, endothelial cells exhibited abnormally elongated morphology which was reversed by 4-PBA pre-treatment (Figure 5D). Quantification of the cellular aspect ratio confirmed that 4-PBA rescued aberrant elongation in Hyperhomocysteinemic cells (Figure 5E). On the contrary, endothelial cells with sub-lethal HHcy showed significant reduction in cellular surface area indicating that cytoplasm of these cells has shrunk and similar to abnormal elongation, this anomaly was also rescued by 4-PBA (Figure 5D,F). Apart from differences in cell morphology, we also noticed that formation of actin stress fibers, which is associated with migratory force generation, were hampered in sub-lethal Hcy treated endothelial cells and 4-PBA pre-treatment caused their reappearance (Figure 5D). Taken together, the above-mentioned data indicate that sub-lethal Hcy-induced adaptive UPR controls endothelial migration defect through aberrant remodeling of the actin cytoskeleton. However, as demonstrated by BrdU incorporation assay, we found that sub-lethal Hcy-induced impaired endothelial proliferation did not improve after pre-treatment with 4-PBA and TUDCA (Figure 5G and Figure S3E). This depicts that adaptive UPR activation influences only the migratory part of sub-lethal HHcy-associated endothelial dysfunction.

### 3.6. Sub-Lethal HHcy Linked Malfunctioned ETC Causes Reduction in Mitochondrial Respiration, a Crucial Regulator of Endothelial Proliferation

**Figure 6:**
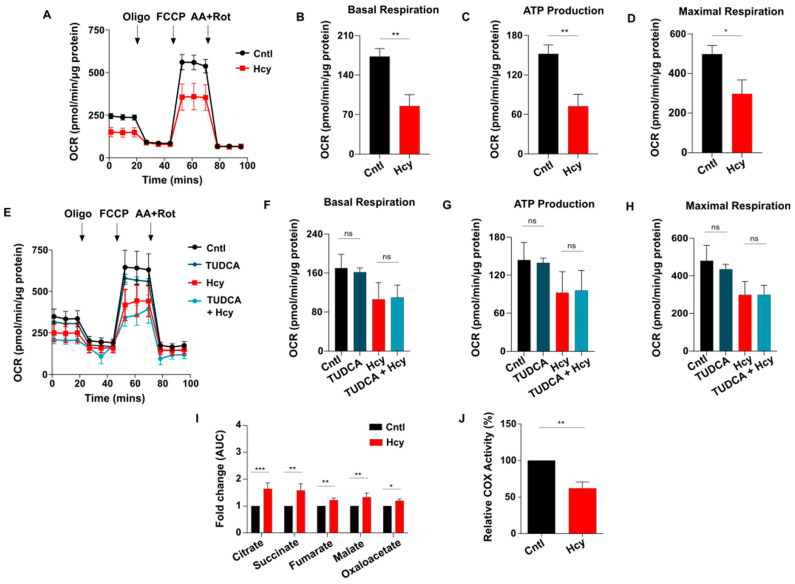
Sub-lethal HHcy linked malfunctional ETC impairs mitochondrial respiration of endothelial cells. (**A**) OCR curves showing drastic reduction in endothelial mitochondrial respiration upon 2 mM Hcy treatment for 24 h as measured by an extracellular flux analyzer. (**B**–**D**) Respective bar graphs demonstrating that in comparison to untreated cells, there is significant decrease in basal respiration, ATP production and maximal respiration of endothelial cells with sub-lethal HHcy. (**E**) OCR curves showing no restoration of sub-lethal HHcy-induced mitochondrial respiration defect in presence of TUDCA. (**F**–**H**) Bar plots respectively showing no significant improvement in the reduction in basal respiration, ATP production and maximal respiration upon TUDCA pre-treatment, compared with sub-lethal Hcy treated endothelial cells. (**I**) Targeted metabolomics mediated quantification exhibiting significant elevation of metabolites of TCA cycle in sub-lethal Hcy treated endothelial cells compared with untreated control cells. AUC, area under the curve. (**J**) Bar plot showing that sub-lethal HHcy in endothelial cells causes significant reduction in enzymatic activity of COX, the terminal electron acceptor of ETC. Data are shown as Mean ± SEM with *n* ≥ 3. * *p* ≤ 0.05, ** *p* ≤ 0.01, *** *p* ≤ 0.001 and ns is non-significant (*p* > 0.05).

Cellular metabolism is a primary mechanism that governs endothelial proliferation [34,35]. Since HHcy itself is a metabolic disorder, we wanted to study its impact on mitochondrial respiration and glycolysis, two major components of endothelial metabolism. Using an extracellular metabolic flux analyzer, we found that oxygen consumption rate (OCR) is drastically reduced in sub-lethal Hcy treated endothelial cells (Figure 6A). Basal respiration, ATP production and maximal respiration, analyzed by measurement of area under the curve (AUC), were decreased by around 40–50% which dictates severe impairment of mitochondrial respiration in endothelial cells exposed to sub-lethal concentration of Hcy (Figure 6B,D). Our next goal was to determine whether along with mitochondrial respiration, sub-lethal HHcy also influences overall mitochondrial function and integrity. Measurement of mitochondrial membrane potential using potentiometric fluorescent dye TMRE showed no significant change in Hyperhomocysteinemic endothelial cells compared with untreated control ones (Figure S4A). Next, analysis of mitochondrial network integrity in mitoGFP transduced cells demonstrated absence of any observable mitochondrial fragmentation and overall mitochondrial network also seemed to be unaltered upon induction of sub-lethal HHcy in endothelial cells (Figure S4B). ImageJ based quantification of mitochondrial length further ascertained no significant change in mitochondrial morphology in Hyperhomocysteinemic endothelial cells (Figure S4C). Together, we demonstrated that while Hcy can impair mitochondrial respiration at a sub-lethal level, overall mitochondrial integrity and health remained unaltered. ER stress by influencing mitochondrial function is known to cause pathological abnormalities in several diseases [59,60]. So, our next objective was to check whether sub-lethal HHcy-induced endothelial mitochondrial respiration defect is a consequence of ER stress-associated adaptive UPR activation. As elucidated by OCR curves and adjoining quantifications, pre-treatment with chemical chaperone TUDCA failed to improve the decrease in mitochondrial respiratory parameters in presence of sub-lethal HHcy (Figure 6E,H). Another chemical chaperone, 4-PBA, produced a similar result (Figure S4D,G). This demonstrates that adaptive UPR is not causal to sub-lethal HHcy-induced endothelial mitochondrial respiration defect. Next, through targeted metabolomics of TCA cycle metabolites we found that sub-lethal Hcy exposure did not cause suppression of TCA cycle either (Figure 6I). Thus, after nullifying ER stress and TCA cycle impairment, we looked into the status of mitochondrial bioenergetics to find out the source of mitochondrial respiration defect involved in sub-lethal HHcy associated endothelial dysfunction. Measurement of cytochrome C oxidase (COX) activity confirmed that sub-lethal HHcy-induced a 40% reduction in the functionality of endothelial mitochondria’s terminal electron acceptor (Figure 6J). Collectively, we demonstrated that despite not affecting overall mitochondrial health, sub-lethally increased Hcy impairs endothelial mitochondrial respiration which happens due to a dysfunctional electron transport chain.

### 3.7. Endothelial Glycolysis Is Elevated upon Sub-Lethal HHcy Mediated Mitochondrial Respiration Defect

**Figure 7:**
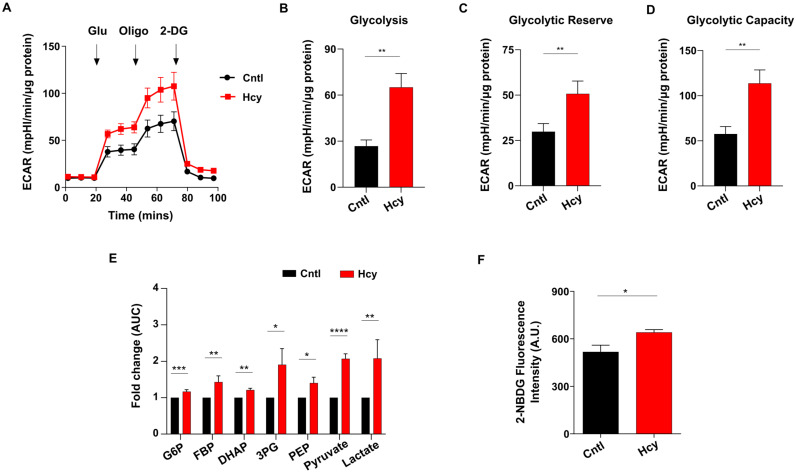
Glycolysis is elevated upon induction of sub-lethal HHcy in endothelial cells. (**A**) ECAR curves showing drastically upregulated glycolysis of endothelial cells treated by 2 mM Hcy for 24 h as measured by an extracellular flux analyzer. (**B**–**D**) Respective bar graphs revealing that in comparison to untreated cells, there is a significant enhancement of glycolysis, glycolytic reserve, and glycolytic capacity of sub-lethal Hcy treated endothelial cells. (**E**) Bar graph of targeted metabolomics showing metabolic intermediates of glycolysis are elevated in endothelial cells with sub-lethal HHcy. AUC, area under the curve. (**F**) Glucose uptake assay using fluorescence analog 2-NBDG showing that in comparison to control cells, consumption of extracellular glucose is higher in sub-lethal Hcy treated endothelial cells. Data are shown as Mean ± SEM with *n* ≥ 3. * *p* ≤ 0.05, ** *p* ≤ 0.01, *** *p* ≤ 0.001, and **** *p* ≤ 0.0001.

Impairment of mitochondrial respiration is known to elevate endothelial glycolysis [34]. As there was a substantial loss of ATP level, we speculated that exposure to sub-lethal dose of Hcy will also increase endothelial glycolysis. Indeed, analysis of extracellular acidification rate (ECAR) showed that glycolysis has sharply increased in Hyperhomocysteinemic endothelial cells (Figure 7A). Quantification of basal glycolysis, glycolytic reserve and glycolytic capacity also revealed that sub-lethal Hcy treated cells had significantly higher levels of these parameters of glycolytic metabolism compared with untreated control cells (Figure 7B,D). To verify this, we analyzed the levels of glycolytic metabolites through targeted metabolomics. In line with our extracellular flux analysis, all the glycolytic intermediates of endothelial cells were found to be significantly elevated upon 2 mM Hcy treatment for 24 h (Figure 7E). To further confirm, we assessed glucose consumption using the fluorescent analog 2-NBDG which established a significantly higher uptake of extracellular glucose in sub-lethal Hcy treated endothelial cells (Figure 7F). Overall, we found that sub-lethal HHcy increases endothelial glycolysis probably to cope with abolished mitochondrial respiration.

### 3.8. Mechanistic Signatures of Moderate HHcy Are Conserved in Adult Endothelial Cells

**Figure 8:**
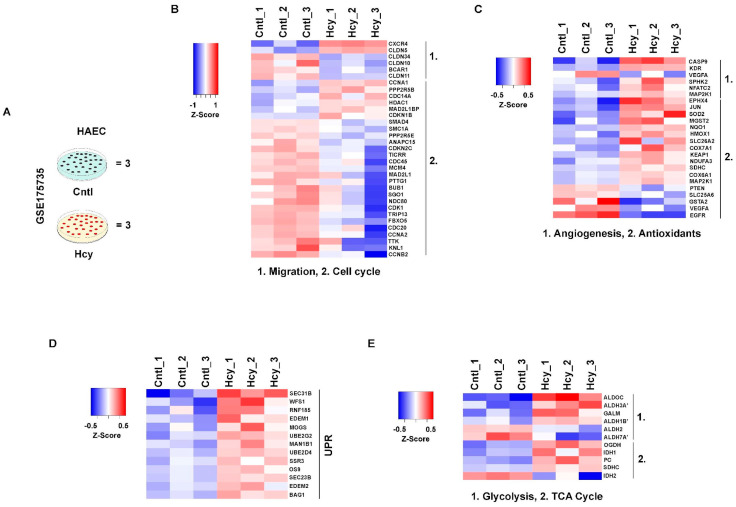
Mechanistic features of pathologically relevant Hcy exposure are conserved in adult endothelial cells. (**A**) Schematic diagram showing experimental model and treatment condition used for microarray profiling of GSE175735 from GEO database, previously published by M Jan et al. (**B**) Heatmap illustrating the trend of downregulation in differentially expressed genes (DEGs) of cell cycle and cellular migration compared between control and Hcy treated (0.5 mM, 48 h) groups. (**C**–**E**) Respective heatmaps showing pathway specific expression of angiogenesis and antioxidant response genes, UPR and metabolism (TCA cycle and glycolysis), in the same Hcy treated and untreated control cell groups. Statistically significant (*p*-value < 0.05) genes were considered to obtain DEGs. Respective color legends representing Z-score ((observed value–mean)/standard deviation) values. Three sets of samples each from control and Hcy treated cells were used for analysis.

Primary and immortalized HUVEC which were used for this study are cellular models of neonatal origin. To check whether adult endothelial cells will respond similarly to a pathologically relevant dose of Hcy, we analyzed a previously published microarray dataset (GSE175735) by M Jan et al., from Gene Expression Omnibus (GEO) in which adult origin primary human aortic endothelial cells (HAEC) were exposed to 0.5 mM Hcy for 48 h [42] (Figure 8A). Our analysis revealed that several genes involved in cellular migration and cell cycle were significantly downregulated upon Hcy treatment (Figure 8B), which is in support of the previous observation by Jan et al. Moreover, affirming our previously mentioned in vitro and in vivo observations, genes associated with angiogenic growth factor signaling showed absence of a trend of downregulation in the Hcy treated group compared with the control group. Similarly, a trend of upregulation was observed in several genes associated with cellular antioxidant defense systems suggesting a possible aversion of oxidative stress even in 0.5 mM Hcy treated (48 h) adult endothelial cells (Figure 8C). Furthermore, we found that a number of genes which belong to UPR are also significantly upregulated in Hyperhomocysteinemic HAEC cells (Figure 8D). To gain insight into cellular metabolism, we then checked differentially altered genetic markers of TCA cycle and glycolysis and observed that in corroboration to our metabolic study, not only TCA cycle but also several markers of glycolysis were exhibiting an upregulated trend upon Hcy treatment (Figure 8E). Overall, as shown in the heat maps, our analysis established that the characteristics of pathological HHcy-induced functional impairments through UPR activation and metabolic alteration, are conserved even in adult endothelial cells.

## 4. Discussion

Endothelial cells constitute the innermost layer of blood vessels and are essential for angiogenesis, a tightly regulated multistep process involving cellular migration and proliferation [61]. Although a large number of clinical and experimental studies have linked HHcy to vascular endothelial dysfunction, it is not clear whether this association is causal or consequential [62,63,64]. Moreover, serum Hcy lowering through vitamin B supplements in patients with cardiovascular complications did not produce encouraging results [65,66]. This in turn suggests that there might be subtle functional changes in Hyperhomocysteinemic endothelial cells that need attention. In this regard, most of the previous cell-based studies focused on severely elevated Hcy mediated endothelial cytotoxicity [7,8,9]. Due to the fact that severe HHcy which is induced by inborn error in Hcy catabolism [46], a rare clinical condition, our aim in this study was to explore the mechanistic basis of how moderate HHcy affects endothelial function in the absence of cell death. For this purpose, we induced HHcy exogenously and generated moderate Hyperhomocysteinemic condition in endothelial cells with no detectable cytotoxicity. Using this cellular model, we found that even at a sub-lethal level, Hcy can cause endothelial dysfunction. Further, our data suggested that sub-lethal increase in Hcy levels can impair endothelial migration and proliferation, two critical components of endothelial function required during angiogenesis. This anti-migratory and anti-proliferative effect of Hcy on endothelial cells was in line with previous studies [22,23,67]. The role of canonical VEGF/VEGFR signaling in the context of HHcy-induced endothelial migration and proliferation impairment are contradictory [26,27,28]. Though the suppression of canonical VEGF signaling has been mostly implicated in angiogenic defects and endothelial dysfunction, several independent angiogenic factors [68] as well as alternative mechanisms beyond VEGF signaling are gaining immense importance recently in this regard [32,69,70]. In our sub-lethal Hcy treated endothelial cells we found that the transcript levels of canonical VEGF/VEGFR signaling markers are not reduced. It indicates the possibility of some other mechanism that may be involved in moderate Hcy-induced endothelial dysfunction beyond the conventional VEGF signaling axis. Several previous reports also suggested that oxidative stress play a pivotal role in inducing cell death in Hyperhomocysteinemic endothelial cells [9,10]. However, we found that moderate HHcy-induced endothelial dysfunction is not associated with excess ROS formation. It can be possible that at moderate HHcy condition, to avoid lethality, endothelial cells maintain an overall ROS balance by intrinsically activating antioxidant defense mechanisms as evident by the increasing trend of expression of major antioxidant enzymes.

Previous research using mammalian model systems of mice [71,72], rats [73,74], and even monkeys [75] had shown that diet-induced HHcy can detrimentally influence the vascular endothelium. As Zebrafish is widely used in modelling cardiovascular research [76], in order to understand whether moderate HHcy can cause vascular abnormality in an animal model also, we generated a zebrafish model of sub-lethal HHcy by targeting CBS. Using this zebrafish model, we found that although sub-lethal HHcy does not influence initial vascular development and viability of the organisms, subtle functional events like angiogenesis of CVP and ISV formation are impaired. This suggests that any specifically altered growth factor signaling may not be the sole cause of sub-lethal HHcy-induced vascular endothelial dysfunction. In a previous study, Lee et al. also reported that exposure to Hcy containing water can induce vascular dysfunction in larval zebrafish [77]. A recent study with a knockout model of HHcy also showed a similar abnormality in ISV formation of zebrafish [78]. Further, to check the specificity of moderate HHcy-induced vascular abnormality in vivo, we also generated a viable zebrafish model of CGL deficiency as control. In humans, CGL deficiency is associated with urinary excretion of excess Cystathionine and may induce cognitive impairment [48]. In this study, we revealed that CGL knockdown does not induce any vascular abnormality which demonstrates that sub-lethal HHcy-induced vascular endothelial dysfunction is not due to an alteration of the transsulfuration pathway per se. It is worth mentioning here that unlike CBS KO mice, CGL deficient mice are viable and do not exhibit any phenotype under normal condition [79]. Lastly, in the line of our cellular data, we found that sub-lethal HHcy-induced vascular endothelial dysfunction in zebrafish is not associated with the suppression of VEGF/VEGFR transcription and ROS induction. 

Hcy is regarded as a potent inducer of ER stress in not only endothelial cells [13,14,16,51] but also in other cell types [52,53]; however, it should be emphasized here that most of the previous studies have connected Hcy-induced ER stress to apoptotic cell death. Our data revealed that at a sub-lethal level also, Hcy can induce ER stress in endothelial cells but it subsequently activates an adaptive UPR that is not terminal in nature and may not necessarily induce cell death. Subsequently by blocking ER stress using chemical chaperones, we unveiled for the first time that in between migration and proliferation defects, sub-lethal Hcy-induced adaptive UPR controls only the migratory axis. The chemical chaperones 4-PBA and TUDCA, which were used in our study, have immense therapeutic potential and produced encouraging results in several clinical trials [80,81]. Being specialized epithelial cells, remodeling of the actin cytoskeleton is absolutely essential for amoeboid migration of endothelial cells [58]. Previous reports showed that upon TNFα treatment, endothelial cells can rapidly undergo cytoskeletal rearrangement and become elongated which ultimately reduces their migration time [82]. Our results confirmed that a similar aberrant modulation of actin cytoskeleton leading to abnormal cell elongation is the mechanistic basis through which ER stress exerts its anti-migratory effect on endothelial cells in moderate HHcy. Previous reports showed that the proteins of the UPR pathways are linked to actin dynamics in other cell types [83,84] which further support our conclusion that adaptive UPR plays an upstream signal to control aberrant cytoskeletal dynamics in endothelial cells that explains their altered migratory phenotype in moderate HHcy.

One of the major determinants of cellular proliferation is metabolism [85]. Particularly, in endothelial cells, the latest research in this aspect suggests that disruption of specific metabolic pathways can impair their proliferation without influencing migratory property [34,35]. Metabolic reprogramming of vascular endothelial cells has also emerged as a novel therapeutic target for pathological angiogenesis in recent years [86,87]. Nevertheless, the regulation of endothelial metabolism in HHcy remains largely unexplored. Our study elucidates that at a sub-lethal level, Hcy can inhibit mitochondrial respiration in endothelial cells. In contrast to previously published data, we revealed that sub-lethal HHcy-induced impairment of mitochondrial respiration may not inevitably cause alteration of mitochondrial membrane potential and fragmentation of mitochondrial network. Diebold et al. reported a similar outcome when endothelial cells were treated with antimycin A, a well-known mitochondrial complex III blocker [34]. ER stress can crosstalk with mitochondria and thereby influence energy metabolism, cell survival, and protein folding which may induce pleiotropic consequences on various diseases like neurodegenerative and lysosomal storage disorders [59,60,88]. However, we observed that mitochondrial respiration defect in moderate HHcy, as assessed in the presence of chemical chaperones, is not a consequence of ER stress-associated adaptive UPR. Our data also suggested that both glycolysis and TCA cycle are upregulated in moderate HHcy. Owing to the fact that TCA cycle intermediates also have anaplerotic roles and can act as precursor for amino acids, fatty acids and nucleotides [89], the role of TCA cycle metabolites in Hyperhomocysteinemic endothelial cells remains an exciting open question. As TCA Cycle which feeds reducing equivalents to mitochondrial respiratory chain, is not repressed upon induction of moderate HHcy in endothelial cells we narrowed down that the electron transport chain itself might be malfunctioning. Indeed, we found that sub-lethal HHcy causes a reduction in the enzymatic activity of COX, the complex IV of ETC. These results are in support of recent observations where complex III and IV deficiency mediated impairment of mitochondrial respiration was reported to be necessary for endothelial function in developmental as well as pathological angiogenesis [34]. It will therefore be interesting to conduct further research focusing on in-depth understanding of the mitochondrial bioenergetics status and its alteration in Hyperhomocysteinemic endothelial cells. Beside mitochondrial respiration, glycolysis also plays a vital role in endothelial cells and its complete suppression can induce cell death [90]. In accordance, our data demonstrated that moderate HHcy rewires glycolysis by upregulating it and that is probably a prerequisite for survival of endothelial cells with compromised mitochondrial respiration in this stressed condition.

Endothelial dysfunction is regarded as an important contributor to the pathobiology of atherosclerosis [91] which is a disease of the adult vasculature. Since fetal tissue derived HUVEC cell line may not fully represent the mature endothelium of the adult vasculature, we used a previously published microarray dataset [42] of adult endothelial cells exposed to Hcy. Our gene expression analysis established that all the mechanistic signatures and processes elucidated in HUVEC cells by us in moderate HHcy are conserved even in endothelial cells of adult origin and that further endorse our experimental observations.

In conclusion, the findings of our work elucidates that in absence of lethality, moderate HHcy can cause endothelial dysfunction by altering their migration and proliferation capacities. Mechanistically, an independent bimodal regulation through adaptive UPR activation and metabolic rewiring act as the upstream cues to generate these defects and that may later on increase the risk of developing vascular diseases. Our results also provide a clue that in contrast to severe HHcy-induced early lethality, the aged or nutritionally deficient population of mild to moderate HHcy can well be clinically vulnerable to the development of cardiovascular diseases with time.

## Data Availability

The transcriptome dataset analyzed in the current study has been previously published by Jan M et al. (2021) [42] and is available at the NCBI Gene Expression Omnibus (accession no. GSE175735). https://www.ncbi.nlm.nih.gov/geo/query/acc.cgi (accessed on 28 March 2023).

## Supplementary Materials

The following supporting information can be downloaded at: https://www.mdpi.com/article/10.3390/cells13030214/s1, Figure S1. Primary HUVEC responds similarly to sub-lethal Hcy treatment. (A) Treatment of primary HUVEC cells with 2 mM Hcy for 24 h did not cause any cytotoxicity as measured by Trypan blue exclusion assay. (B) Bar plot showing that in presence of proliferation blocker Mitomycin C, migration of primary endothelial cells is drastically reduced upon sub-lethal Hcy treatment. (C) Percentage of proliferating cells, determined by BrdU cell proliferation assay, is significantly less in sub-lethal Hcy treated primary HUVEC compared with untreated control cells. Data are shown as Mean ± SEM with n ≥ 3. * *p* ≤ 0.05, **** *p* ≤ 0.0001 and ns is non-significant (*p* > 0.05); Figure S2. Sub-lethal HHcy induces abnormality in cardiac structure and function in vivo. (A) Representative brightfield images showing induction of severe pericardial edema in CBS MO injected embryos at 4 dpf. Arrow indicating enlarged pericardial area of CBS morphants. Scale bar, 0.1 mm. (B) Bar graph confirming pericardial area of CBS MO injected embryos is significantly higher compared with scrambled MO injected embryos at 4 dpf. (C) Bar graph showing that in comparison to SC MO injected embryos, heartbeat of CBS morphants is significantly reduced. Data are shown as Mean ± SEM with n ≥ 3. * *p* ≤ 0.05 and ** *p* ≤ 0.01; Figure S3. Effect of chemical chaperone mediated rescue of ER stress in sub-lethal HHcy treated endothelial cells. (A,B) Representative western blot showing sub-lethal Hcy treatment-induced aberrant upregulation of GRP78 expression was rescued upon pre-treatment with 4-PBA (1 mM) and TUDCA (1 mM), respectively. As a loading control β-actin was used. Accompanying bar plots showing densitometric analysis (normalized to β-actin) of the protein bands. (C,D) Scratch wound assay images depicting that at 24 h in presence of proliferation blocker Mitomycin C, sub-lethal Hcy treatment-induced endothelial migration defect is rescued upon pre-treatment with 4-PBA (1 mM) and TUDCA (1 mM), respectively. (E) Bar plot of BrdU cell proliferation assay showing no beneficial effect of 4-PBA (1 mM) on impairment of endothelial proliferation-induced by 2 mM Hcy treatment at 24 h. Data are shown as Mean ± SEM with n ≥ 3. * *p* ≤ 0.05, ** *p* ≤ 0.01, **** *p* ≤ 0.0001 and ns is non-significant (*p* > 0.05); Figure S4. Sub-lethal HHcy-induced respiration defect neither influence mitochondrial health nor is it an outcome of ER stress. (A) Bar plot representing no alteration of mitochondrial membrane potential upon 2 mM Hcy treatment for 24 h, as measured using potentiometric dye TMRE. An uncoupler CCCP was used as a positive control. (B) Representative confocal images of mitoGFP transduced HUVEC/TERT2 cells showing that sub-lethal Hcy treatment did not alter integrity of mitochondrial network of endothelial cells. Scale bar, 10 μm. (C) Measurement of mitochondrial length by ImageJ analysis exhibiting no significant difference in between Hcy treated and non-treated cells. (D) OCR curves showing no improvement of sub-lethal Hcy treatment-induced mitochondrial respiration defect in presence of chemical chaperone 4-PBA (1 mM). (E–G) Respective bars of basal respiration, ATP production and maximal respiration revealing no improvement upon 4-PBA (1 mM) pre-treatment compared with only Hcy treated endothelial cells. Data are shown as Mean ± SEM with n ≥ 3. * *p* ≤ 0.05 and ns is non-significant (*p* > 0.05); Table S1: List of Primers used in this study for qPCR analysis; Table S2: Optimized parameters of different metabolites analyzed through targeted metabolomics.
