# Supplementary material for: Moderate Elevation of Homocysteine Induces Endothelial Dysfunction through Adaptive UPR Activation and Metabolic Rewiring
Source: Cells. 2024 Jan 24;13(3):214. doi: 10.3390/cells13030214 (PMC10854856; doi:10.3390/cells13030214)
Supplement: Supplementary file 1 [file cells-13-00214-s001.zip › cells-2731405-supplementary .pdf]

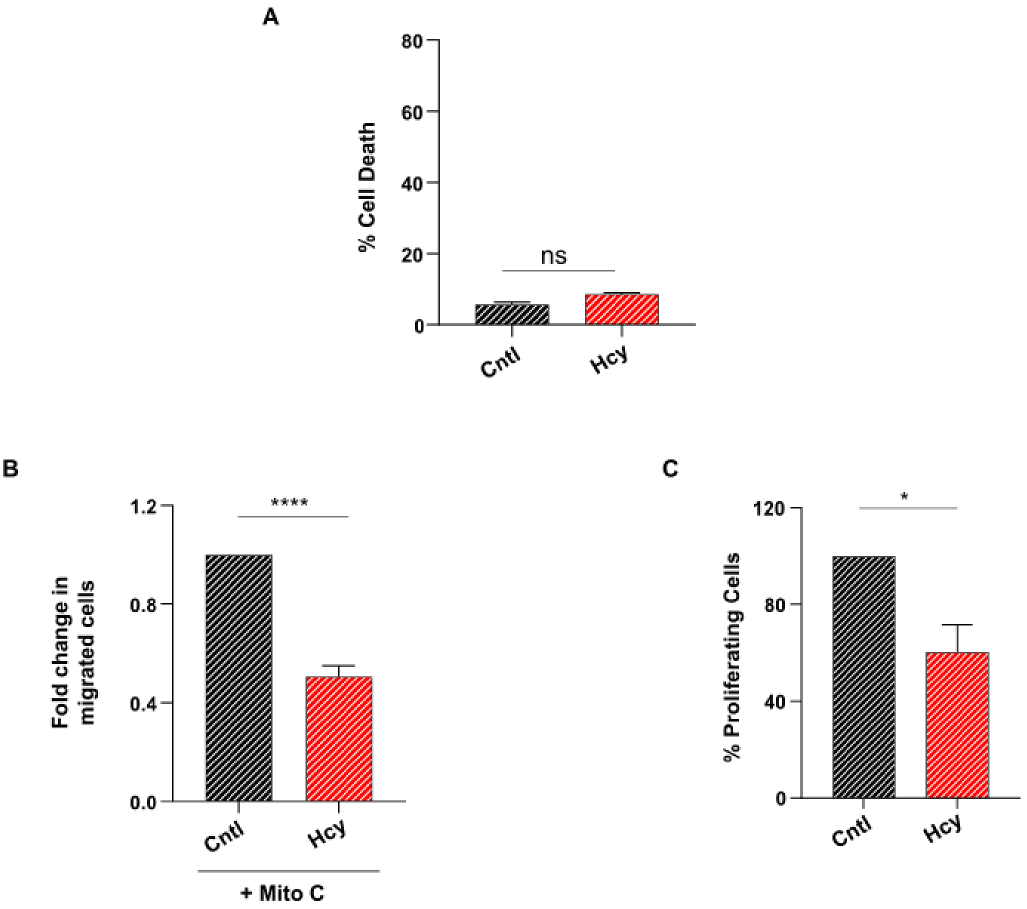

Figure S1. Primary HUVEC responds similarly to sub-lethal Hcy treatment. (**A**) Treatment of primary HUVEC cells with 2 mM Hcy for 24 h did not cause any cytotoxicity as measured by Trypan blue exclusion assay. (**B**) Bar plot showing that in presence of proliferation blocker Mitomycin C, migration of primary endothelial cells is drastically reduced upon sub-lethal Hcy treatment. (**C**) Percentage of proliferating cells, determined by BrdU cell proliferation assay, is significantly less in sub-lethal Hcy treated primary HUVEC compared to untreated control cells. Data are shown as Mean±SEM with n ≥ 3. *P≤0.05,****P≤0.0001 and ns is non-significant (P>0.05).

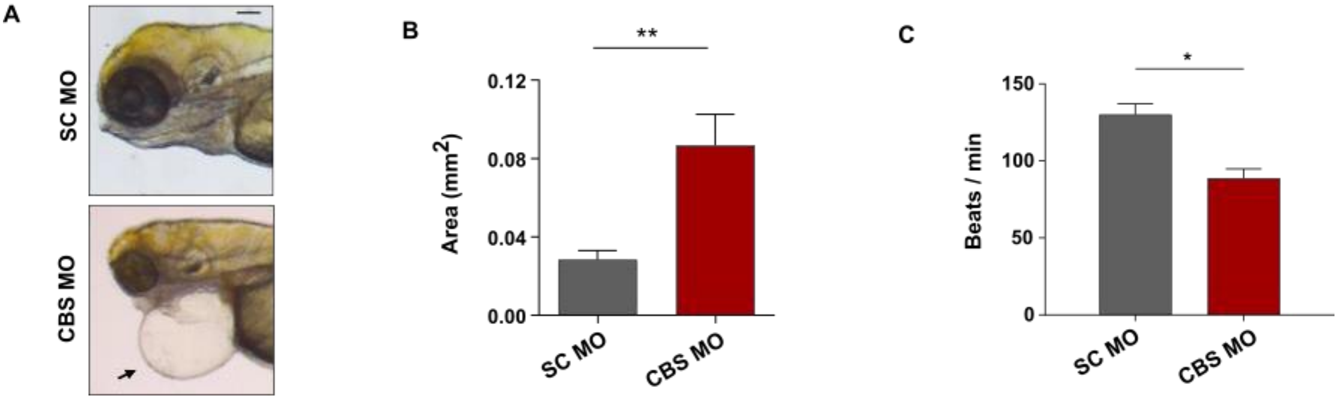

Figure S2. Sub-lethal HHcy induces abnormality in cardiac structure and function *in vivo*. (**A**) Representative brightfield images showing induction of severe pericardial edema in CBS MO injected embryos at 4 dpf. Arrow indicating enlarged pericardial area of CBS morphants. Scale bar, 0.1 mm. (**B**) Bar graph confirming pericardial area of CBS MO injected embryos is significantly higher compared to scrambled MO injected embryos at 4 dpf. (**C**) Bar graph showing that in comparison to SC MO injected embryos, heartbeat of CBS morphants is significantly reduced. Data are shown as Mean±SEM with n ≥ 3. *P≤0.05 and **P≤0.01.

Figure S3

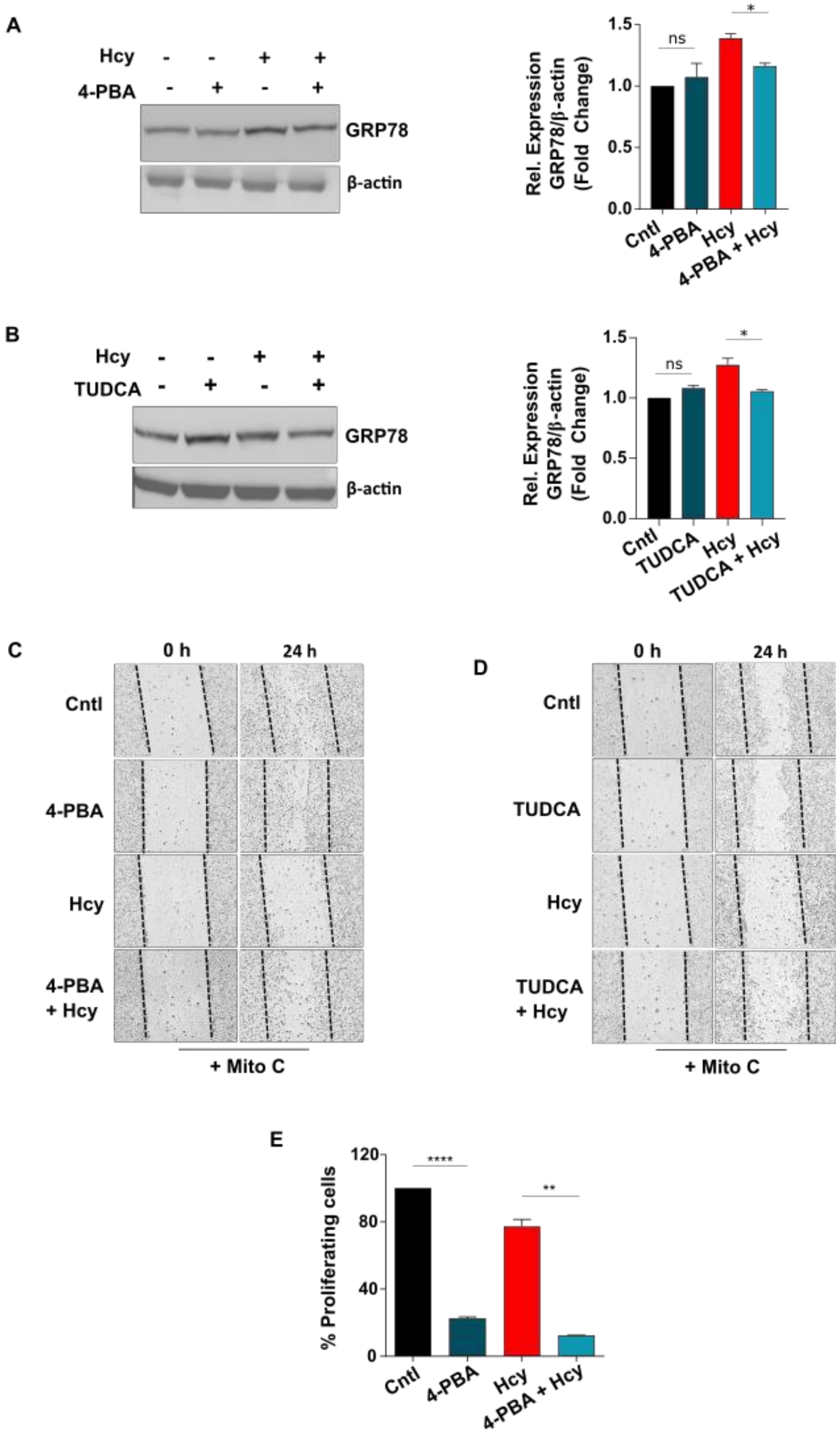

A
Hcy - - + +
4-PBA - + - +
GRP78
β-actin
Rel. Expression GRP78/β-actin (Fold Change)
1.5
1.0
0.5
0.0
ns
*
Cntl
4-PBA
Hcy
4-PBA + Hcy
B
Hcy - - + +
TUDCA - + - +
GRP78
β-actin
Rel. Expression GRP78/β-actin (Fold Change)
1.5
1.0
0.5
0.0
ns
*
Cntl
TUDCA
Hcy
TUDCA + Hcy
C
0 h
24 h
Cntl
4-PBA
Hcy
4-PBA + Hcy
+ Mito C
D
0 h
24 h
Cntl
TUDCA
Hcy
TUDCA + Hcy
+ Mito C
E
% Proliferating cells
120
80
40
0
****
**
Cntl
4-PBA
Hcy
4-PBA + Hcy

Figure S3. Effect of chemical chaperone mediated rescue of ER stress in sub-lethal HHcy treated endothelial cells. (**A**) & (**B**) Representative western blot showing sub-lethal Hcy treatment induced aberrant upregulation of GRP78 expression was rescued upon pre-treatment with 4-PBA (1 mM) and TUDCA (1 mM), respectively. As a loading control β-actin was used. Accompanying bar plots showing densitometric analysis (normalized to β-actin) of the protein bands. (**C**) & (**D**) Scratch wound assay images depicting that at 24 h in presence of proliferation blocker Mitomycin C, sub-lethal Hcy treatment induced endothelial migration defect is rescued upon pre-treatment with 4-PBA (1 mM) and TUDCA (1 mM), respectively. (**E**) Bar plot of BrdU cell proliferation assay showing no beneficial effect of 4-PBA (1 mM) on impairment of endothelial proliferation induced by 2 mM Hcy treatment at 24 h. Dataare shown as Mean±SEM with n ≥ 3. *P≤0.05, **P≤0.01, ****P≤0.0001 and ns is non-significant (P>0.05).

Figure S4

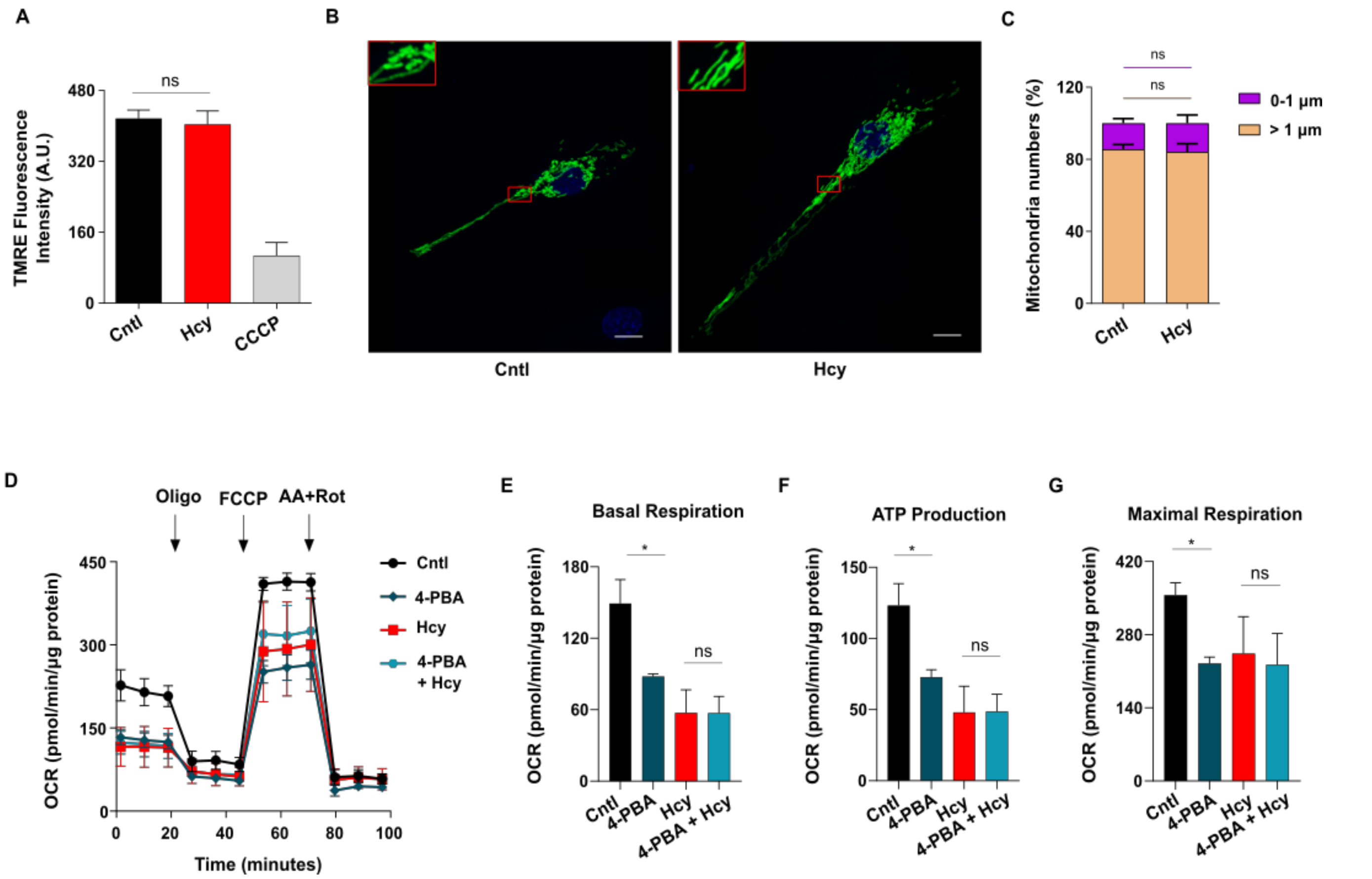

Figure S4. Sub-lethal HHcy induced respiration defect neither influence mitochondrial health nor is it an outcome of ER stress. (**A**) Bar plot representing no alteration of mitochondrial membrane potential upon 2 mM Hcy treatment for 24 h, as measured using potentiometric dye TMRE. An uncoupler CCCP was used as a positive control. (**B**) Representative confocal images of mitoGFP transduced HUVEC/TERT2 cells showing that sub-lethal Hcy treatment did not alter integrity of mitochondrial network of endothelial cells. Scale bar, 10 µm. (**C**) Measurement of mitochondrial length by ImageJ analysis exhibiting no significant difference in between Hcy treated and non-treated cells. (**D**) OCR curves showing no improvement of sub- lethal Hcy treatment induced mitochondrial respiration defect in presence of chemical chaperone 4-PBA. (1 mM). (**E**), (**F**) & (**G**) Respective bars of basal respiration, ATP production and maximal respiration revealing no improvement upon 4-PBA (1 mM) pre-treatment as compared to only Hcy treated endothelial cells. Data are shown as Mean±SEM with n ≥ 3. *P≤0.05 and ns is non-significant (P>0.05).

Table S1. List of Primers used in this study for qPCR analysis.

Gene	Primer sequence
Zebrafish - VEGFAA - forward	GCCCACATACCCAAAGAAGG
Zebrafish - VEGFAA - reverse	CTCATCGGGATACTCCTGGAT
Zebrafish - VEGFR2 - forward	TTTGGTAGAGGGATCTCGTC
Zebrafish - VEGFR2 - reverse	GCGTACCGATGACACATTTC
Zebrafish - VEGFR1 - forward	ATGGGAACAGCAGCACTCTT
Zebrafish - VEGFR1 - reverse	TGAAGACGGAGGGACAATC
Zebrafish - 18S - forward	TCGCTAGTTGGCATCGTTTATG
Zebrafish - 18S - reverse	CGGAGGTTCGAAGACGATCA
Human - VEGFA - forward	TCCAACTTCTGGGCTGTTCT
Human - VEGFA - reverse	CCCCTCTCCTCTTCCTTCTC
Human - VEGFR2 - forward	TGGGGATTGACTTCAACTGG
Human - VEGFR2 - reverse	TTCTTGGTCATCAGCCCACT
Human - VEGFR1 - forward	ACCACGCCCAGTCAAATTAC
Human - VEGFR1 - reverse	TGGGAATTGCTTTGGTCAAT
Human - 18S - forward	CTACCACATCCAAGGAAGCA
Human - 18S - reverse	TTTTTCGTCACTACCTCCCCG

Table S2. Optimized parameters of different metabolites analyzed through targeted metabolomics.

Sr No.	Metabolite	Precursor (m/z)	Fragment (m/z)	Peak type	Charge	Collision Energy (V)
1	Pyruvate	87	42.99	Quantifier	-1	10
2	Lactate	89	42.99	Quantifier	-1	20
			40.99	Qualifier	-1	
3	Fumarate	115	71.01	Quantifier	-1	20
			44.49	Qualifier	-1	
4	Succinate	117.01	99	Qualifier	-1	20
			73.01	Quantifier	-1	
5	Oxaloacetate	130.99	87	Quantifier	-1	20
			59	Qualifier	-1	
6	Malate	133.01	114.9	Quantifier	-1	10
			72.98	Qualifier	-1	
			71.0	Qualifier	-1	
7	Phospho- enol pyruvate (PEP)	166.97	78.94	Quantifier	-1	10
8	Dihydroxyacetone phosphate (DHAP)	168.98	96.95	Qualifier	-1	20
			78.94	Quantifier	-1	
9	3-Phosphoglycerate (3PG)	184.98	96.95	Qualifier	-1	20
			78.94	Quantifier	-1	
10	Citrate	191.01	110.9	Quantifier	-1	20
			86.99	Qualifier	-1	
11	Glucose-6-phosphate	259.02	138.9	Qualifier	-1	20
			96.95	Quantifier	-1	
			78.94	Qualifier	-1	
12	Fructose-1,6- bisphosphate	338.98	96.95	Quantifier	-1	20
			78.94	Qualifier	-1	
